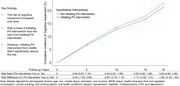# Physical Activity from Midlife and Incident Cognitive Impairment: Whitehall II Study 1997‐2016

**DOI:** 10.1002/alz70860_103019

**Published:** 2025-12-23

**Authors:** Yanan Zhang, Anwar T Merchant, Jingkai Wei

**Affiliations:** ^1^ University of South Carolina, Columbia, SC, USA; ^2^ University of Texas Health Science Center at Houston, Houston, TX, USA

## Abstract

**Background:**

Physical activity (PA) from midlife may be beneficial for reducing cognitive impairment. While the progress of cognitive decline starts from midlife, potential PA intervention may need to be initiated from midlife. However, randomized controlled trials (RCTs) of PA and cognitive outcomes are often conducted among older adults ≥65 years. While RCTs of PA initiated from midlife for decades are not feasible, target trial emulation provides a new approach to estimate the effectiveness using existing observational data. We aimed to emulate a target trial to estimate the effectiveness of PA initiated from midlife in reducing cognitive impairment through late life.

**Method:**

Participants of the Whitehall II study aged 45‐64 years in 1997‐1999, free of cognitive impairment, were included in the analysis. Hypothetical PA intervention was defined as meeting the guideline of ≥150 minutes per week of moderate‐to‐vigorous PA, based on the responses to the Minnesota leisure‐time PA Questionnaire. Incident cognitive impairment was defined as the Mini Mental State Examination score <24 or 1.5 standard deviations below the mean on other cognitive tests (20‐word free recall test, Alice Heim 4‐I test, Mill Hill vocabulary test, phonemic and semantic test). Participants were followed till cognitive impairment, loss‐to‐follow‐up or end of study phase (2015‐2016). Parametric g‐formula was used to estimate the effectiveness. The risk of cognitive impairment was estimated for the entire cohort (1) initiating hypothetical PA from midlife, and (2) not initiating hypothetical PA intervention from midlife and throughout the follow‐up period. Risk ratios and risk differences were also calculated, while controlling for confounding and loss to follow‐up. 95% confidence intervals were estimated using Monte Carlo simulations.

**Result:**

A total of 3,309 participants (26% female, all White) were included in the analysis. The cumulative risk of cognitive impairment increased over time. After 18 years, initiating hypothetical PA intervention from midlife onwards did not show significant reduction of cognitive impairment.

**Conclusion:**

Maintaining recommended PA levels from midlife did not show significant improvement in cognitive impairment. Future studies are expected to estimate the effectiveness of alternative strategies for long‐term PA intervention from midlife in reducing cognitive outcomes.